# Bipolar Stigma in Jewish Communities in the United States

**DOI:** 10.1192/j.eurpsy.2022.312

**Published:** 2022-09-01

**Authors:** L. Smith, K. Brewer, R. Gearing, L.C. Carr, D. Clark, A. Robinson, D. Roe

**Affiliations:** 1 University of Houston, Social Work, Houston, United States of America; 2 University of New Hampshire, College Of Human And Health Sciences, Durham, United States of America; 3 University of Houston, Mh-rites Research Center, Houston, United States of America; 4 University of Haifa, Community Mental Health, Haifa, Israel

**Keywords:** BIPOLAR, Public Stigma, Mood disorders, Jewish

## Abstract

**Introduction:**

This study investigated differences in mood disorder public stigma endorsed by Jewish adults. Specifically, it examined the association between public stigma and the symptomatology and gender of individuals with mood disorders and characteristics of respondents. The symptomatology investigated included major depressive disorder and bipolar disorder presenting with mania or depression. The public stigma factors measured for mood disorders were recovery, relationship disruption, hygiene, anxiety, and treatment/professional efficacy.

**Objectives:**

Do symptomatology and gender predict stigma for mood disorders? For Jewish adults, do gender, age, religious characteristics, mental health history, and perceived stigma for mental illness predict their stigma toward individuals with mood disorders?

**Methods:**

A convenience sample of 243 Jewish adults were randomly administered vignettes using a factorial design. MANCOVA was used for analysis. The Mental Illness Stigma Scale (Day et al., 2007) and the Devaluation of Consumer scale (Struening et al., 2001) were used to measure public and perceived stigma respectively.

**Results:**

showed that recovery, relationship disruption, and hygiene stigmas were associated with vignette subject symptomatology, an interaction was found between respondent gender and age for treatability/professional efficacy stigma, and perceived stigma was correlated with public stigma factors. Consistent with previous research, the highest levels of stigma were found for individuals with bipolar disorder presenting with mania (Wolkenstein & Meyer, 2008).

**Conclusions:**

These findings increase our knowledge of mood disorder stigma existing in the Jewish community and supports research showing that bipolar disorder presenting with mania is the most stigmatized type of mood disorder symptomatology (Wolkenstein & Meyer, 2008).

**Disclosure:**

No significant relationships.

